# Determinant factors in adopting mobile health application in healthcare by nurses

**DOI:** 10.1186/s12911-022-01784-y

**Published:** 2022-02-22

**Authors:** Soghra Nezamdoust, Mohammadhiwa Abdekhoda, Azad Rahmani

**Affiliations:** 1 School of Health Management and Medical Informatics, Tabriz University of Medical Sciences, Tabriz, Iran; 2 Department of Medical Library and Information Sciences, School of Health Management and Medical Informatics, Tabriz University of Medical Sciences, Tabriz, Iran; 3 Department of Medical Surgical Nursing, Faculty of Nursing and Midwifery, Tabriz University of Medical Sciences, Tabriz, Iran

**Keywords:** Mobile applications, Nursing informatics, Technology Acceptance Model (TAM), Diffusion of Innovations (DOI), Nurses

## Abstract

**Background:**

Mobile applications are among effective learning tools and have a significant role in transferring information and knowledge to nurses. The current study was carried to identify the factors affecting nurses’ use of practical health related mobile applications in education and patient interaction based on the combined Technology Acceptance Model (TAM) and Diffusion of Innovation (DOI).

**Method:**

The study is a descriptive-analytical study with a cross-sectional method. The research population includes nurses working at Tabriz University of Medical Sciences hospitals, 150 of which were selected as the research sample using simple and available sampling. The data collection instrument was a questionnaire, the validity and reliability of which were confirmed (α = 0.9). Data analysis was carried out using a correlation test and regression analysis by applying SPSS _v16_ software.

**Results:**

The findings show that perceived usefulness and perceived ease of use have a direct and significant effect on the rate of using mobile applications by nurses (*P* value ≤ 0.01), [(β = 0.52), (β = 0.40)]. Other findings indicate that relative advantage, compatibility, trialability and observability, have a direct and significant effect on nurses’ use of mobile applications, while complicatedness does not have a significant effect.

**Conclusion:**

The current study identifies the effective factors in nurses’ use of health-related mobile applications based on an integrated model of TAM and DOI. Designers of mobile applications should consider these factors in designing and developing programs so that mobile applications can successfully fulfill their purpose in healthcare.

**Supplementary Information:**

The online version contains supplementary material available at 10.1186/s12911-022-01784-y.

## Introduction

Among health personnel, nurses have a direct responsibility in maintaining the health of individuals in society and are committed to closer relations and numerous important roles such as clinical care, consultation, follow-up of accurate treatment, and teaching prevention methods [1]. Nurses are the biggest group of health service providers and play a significant role in teaching patients; whereas they have better access to patients and their families and spend much time caring for them [2, 3]. Continuous scientific advances along with patients varying situations requires nurses, as the biggest group of health service providers, to identify patients’ problems by combining their technical skills and professional knowledge to reduce error and increase the quality of care by designing, implementing and evaluating the program. Recently, it is observed that nurses who base their performance on authentic health information are able to make better decisions and provide higher quality care [4].

Smartphones, like any computer, have software and various practical applications. In the smartphone software market, the term “application” or App for short, has become more well-known than software, and this term has the meaning of practical software, which is known as smartphone applications or smartphone software. One of the methods used today to obtain health information are practical mobile applications, which are being used by people more than ever before due to their easy access, in order to easily and quickly obtain health information compared to other sources. For this means, numerous companies are making efforts to provide practical mobile applications with various usages for different groups of people.

Until now, various research studies have been carried out with regard to nurses’ use of mobile applications. For example, O’Connor et al. [5] studied the student' perspective concerning adopting smartphones and mobile applications in clinical nursing education. Hsu et al. [6] conducted a qualitative study to review the nursing student's experiences of using a smart phone application for physical assessment course. Mazaheri et al. [7] examined the effect of clinical training using mobile phone of medication errors of nursing trainees. Append the details of each articles have been presented in Additional file 1: supplementary file.

Nurses use mobile health applications for various reasons, as these applications have a considerable potential in enhancing their professional activities. Several studies have reported that nurses use these applications to access evidence-based scientific knowledge, support evidence-based decision-making, enhance performance skills of nursing students, and for problem-based methods of teaching (Johansson et al. 2013; Jindal et al. 2018, Choi et al. 2018; Thoma-Lurken et al. 2019; Ferguson et al. 2019). In addition, it is observed from the literature that these applications are used by nurses to support their daily workflow, data entry, birth data tracking and analysis, documentation practices, for the hospital Electronic Health Record (EHR), in order to facilitate documentation at the bedside, for integrated management of chronic conditions, for longitudinal health data, to manage data for the care of mothers, patient information management, personalization, to provide an easy input interface and various outputs for nursing records, and support documentation at the bedside by means of a user-centered approach [8–13]. Another considerable aspect for the use of mobile health applications by nurses is health promotion. Nurses make use of these programs to track patients’ health indicators such as physical activity, diet and sleep and as a guided approach for promoting health and healing in primary health care settings [10, 14, 15]. Therefore, the adoption of mobile health applications is undoubtedly necessary for nurses, as the victory or defeat of medical services is dependent on it. Nurses are among the medical staff most directly responsible for maintaining the health of individuals and are in close contact with their patients. As a result, they have various important roles that require providing clinical care, consultation, follow-up of accurate treatment and teaching disease prevention methods [1]. While nurses comprise the largest group of health care providers and play a significant role in the continuity of healthcare, they have a major role in improving and maintaining health at various levels of the healthcare system [1]. Meanwhile, having such a key role in the healthcare system, they have a valuable role in educating patients since they have better access to patients and their families and devote a great amount of time caring for them [2, 3].

Nevertheless, as previously mentioned, not all applications have been successful and still render worries, such as compatibility with the nurses’ job and concerns regarding privacy and safety [16]. From another perspective, a crucial step to comprehensive adoption of mobile applications is to identify determinant factors in adopting mobile health applications by nurses and pinpoint the numerous advantages that it brings forth.

Electronic learning is a type of learning provided by means of various electronic tools such as smartphones and has advantages such as access at any time and place, reduced educational costs, flexibility, with an effective and satisfactory learning process. In addition, it results in enhancing a sense of responsibility and being self-regulatory, having self-evaluation, critical thinking, and independent and in-depth thinking [17, 18]. Also, among other advantages of e-learning using smartphones are an increase in learning quality and learners’ gain, easy accessibility to a large amount of information, quick and timely access to information in a short time, reduction in some educational costs, enhanced quality, precision and accuracy of content in addition to the scientific advancement of students and teachers [19, 20].

## Hypotheses

Considering the factors that affect nurses’ use of smartphone applications is a significant issue and sufficient research studies have not been conducted in this realm thus far. With regard to the effective factors in using and applying smartphone health applications by nurses’ in this field, this study was carried out with the aim of identifying effective factors in nurses’ use of smartphone health applications integrated with two models of DOI and TAM. Davis presented the Technology Acceptance Model (TAM) in 1998 [21]. The main factors of Davis’s Technology Acceptance Model are: (1) perceived usefulness and (2) perceived ease of use. The rate of consumer’s perception regarding a new product or service, different and better than alternative products and services, is known as the relative advantage or perceived usefulness. Ease of use is referred to as the measurement of user expectation when working with a system without added effort [22].

The Diffusion of Innovations (DOI) is a theory related to how and why a new idea is spread in organizations and cultural and social networks and was presented by Rogers [23]. Rogers considers a number of characteristics for the essence of innovation. From his standpoint, these characteristics are effective in the innovation reliability coefficient on the part of the individual. (1) Relative advantage: the rate that an innovation seems more adequate and is visualized in the individuals mind compared to previous options. (2) Compatibility: the rate that an innovation is considered compatible with common values, previous experiences and potential needs of the consumer. (3) Complicatedness: the rate that an innovation is visualized in the individuals mind whereas its perception and use is not so complex. (4) Trialability: the rate that an innovation has the possibility of experimentation and trial and error. (5) Observability: the rate at which the results and outcome of an innovation are observable and tangible for others [22].

From a combination of the two theories, TAM and DOI, we obtain an integrated framework to better identify and predict user acceptance and enhance the adoption of technological innovation. Among the common features of both theories are organizational background, technologies and individual characteristics [24]. A collection of innovation features was verified afterwards based on extreme similarities between TAM and DOI. In addition, an accurate analysis of TAM indicates that this model has considerable capacities for being integrated in the developed model such as DOI for better perception of individuals’ behavior in the acceptance and adoption of the new technology [22]. Studying Nursing's behavior in adopting mobile health application is multidimensional phenomenon and need a both strong theoretical and analytical model to examine. As claimed by literature TAM and DOI, both are fundamental and well-fixed models and theories, and have considerable capacity to elucidate the determinant factors in adopting and diffusion of new technology. Altogether, studying nurses’ usage of mobile health applications can effect of integration of DOI and TAM and presented a innovative and comprehensive model to elucidate end- user's behavior in adopting new technologies.

With regard to the explanation given, and the proposed conceptual model of this study, the following hypotheses are provided.

Perceived usefulness, defined as “the degree to which an individual believes that using a particular system will enhance his or her job performance,” is related to job effectiveness, productivity, and the relatively significant role of systems in a user’s job. Meanwhile, perceived ease of use is known as “the degree to which an individual believes that using a particular system is free of effort” [24]. It is obtained from the literature that perceived usefulness and perceived ease of use are essential in healthcare practitioners’ intention for accepting and using information systems [25]. Hence, the following hypotheses were put forth:

### H1

Perceived usefulness has an effect on the nurses’ use of smartphone applications.

### H2

Perceived ease of use has an effect on the nurses’ use of smartphone applications.

The research findings revealed that perceived ease of use could influence the new technology adoption through perceived usefulness and should be considered as a determining factor in new technology acceptance as stated in the following hypothesis:

### H3

Perceived ease of use has a direct effect on perceived usefulness.

Rogers defines relative advantage as “the degree to which an innovation such as electronic medical record (EMR) is perceived as superior to the innovation it supersedes” [22]. A number of studies have implied that relative advantage should be considered in comprehensive adoption of new technology (Wu 2008; Zhang 2008; Abdekhoda 2016; Ping Yu 2009; Conrad 2009), thus yielding the fourth hypothesis H4:

### H4

Relative Advantage has a direct effect on perceived usefulness.

Compatibility is defined as “the degree to which an innovation is perceived as consistent with the individual’s existing values, beliefs, past experiences, and needs” [22]. From a review of the literature, it was found that compatibility is a determinant factor in adopting new technologies [22, 26, 27], therefore, hypotheses H5 and H6 were presented as follows:

### H5

Compatibility has a direct effect on perceived usefulness.

### H6

 Compatibility has a direct effect on perceived ease of use.

According to Rogers, complicatedness is defined as “the degree to which an innovation is perceived as relatively difficult to understand and use”. Clearly speaking, complicatedness should consider the dilemma posed by ease of use, as recognized in many of the behavioral intent models such as TAM [22]. Thus, H7 and H8 were put forth as:

### H7

Complicatedness has a direct effect on perceived usefulness.

### H8

Complicatedness has a direct effect on perceived ease of use.

A number of studies approve the positive and significant effect of observability on perceived ease of use [28]. Based on the literature, observability is “the degree to which the results of an innovation are visible to others. Innovation outcomes that are easily observed tend to be adopted more quickly than those with more subtle outcomes [29]. Hence, hypothesis H9 is presented as:

### H9

Observability has a direct effect on perceived ease of use.

Finally, Rogers defined trialability as “the degree to which an innovation will be available for trial usage before adoption” [22]. It has been concluded from some studies that trialability has a direct and considerable effect on perceived ease of use which is crucial and should be further considered [30–33]. Thus, Hypothesis 10 is presented as:

### H10

Trialibility has a direct effect on perceived ease of use.

**Fig. 1:**
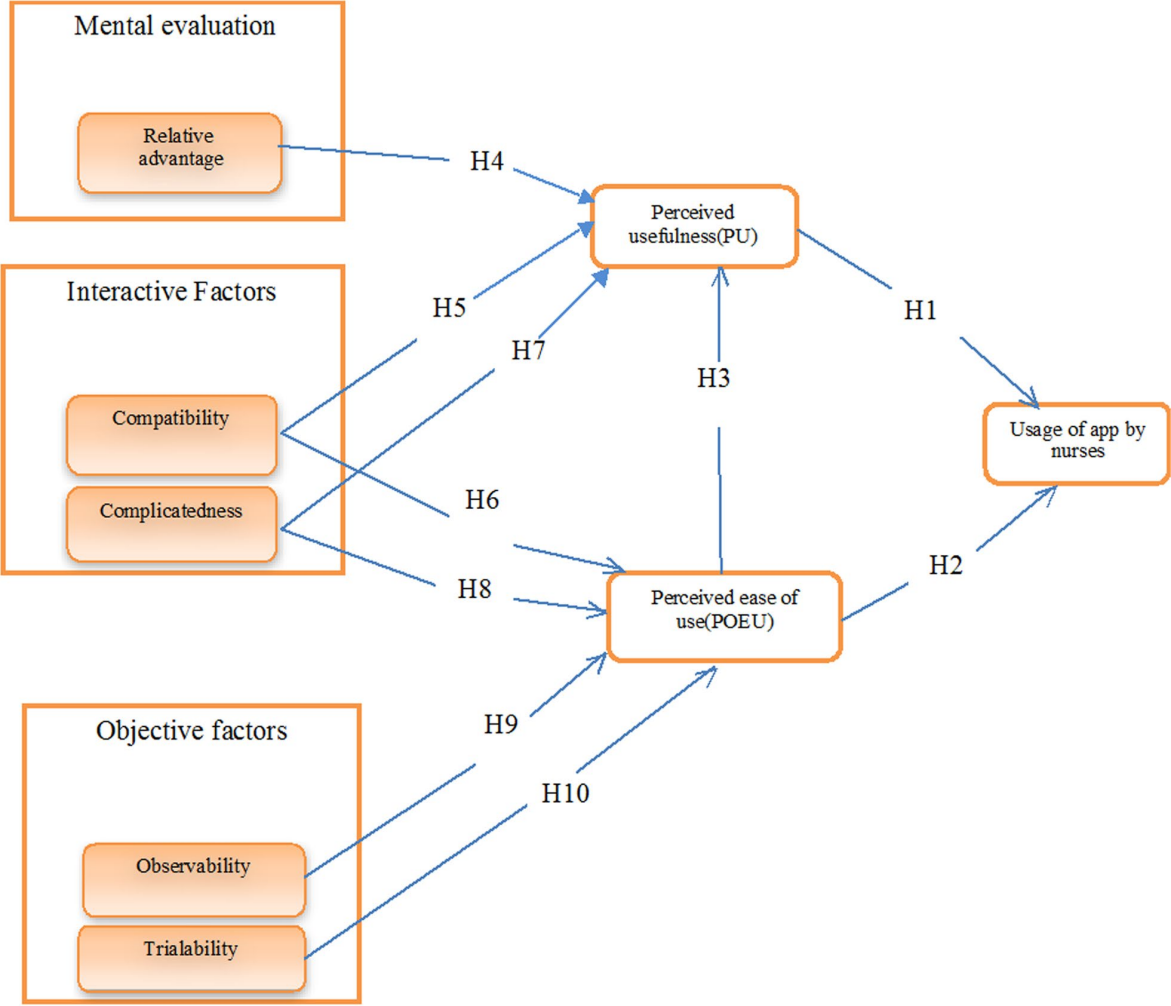
Hypotheses and the proposed integrated model of TAM–DOI

A summary of the hypotheses and the proposed integrated model are given in Fig. 1.

## Methods

The current study is a descriptive-analytical study with a cross-sectional method. The aim of this study is to identify effective factors in nurses’ use of smartphone health applications based on the integrated model of TAM and DOI. This study was carried out at educational hospitals affiliated to Tabriz University of Medical Sciences.

All nurses working at educational hospitals and treatment centers of Tabriz University of Medical Sciences were selected as the research population. Data analysis was conducted using Structural Equation Modeling (SEM) and the sample was selected based on the SEM technique. As put forth by Tanaka in 1987, the sample size is a controversial issue in structural equation modeling [16]. The sample size for each item of the questionnaire for existing topics was considered between 3 and 5. Finally, a number of 150 participants among the nurses working at Tabriz University of Medical Sciences were randomly selected as the research sample. The inclusion criteria are use of smartphone applications and tendency to cooperate with the study and fill out the questionnaire.

As the data collection tool, a researcher-made questionnaire was adapted based on studies Wilkins (2008), Nair (2011), Morton (2008), Conrad (2009), Abdekhoda (2016), and Asadi (2020) [22, 30, 34–37]. The questionnaire contains 9 sections, including demographic information, TAM model factors, perceived usefulness and perceived ease of use; and the DOI model, including 5 indices of relative advantage, compatibility, complicatedness, observability, and trialability. Survey questions used to measure the constructs of TAM –DOI are shown in Table 1.

**Table 1:** Survey questions used to measure the constructs of TAM–DOI

Construct	Item number	Item
Perceived usefulness	1	Mobile applications enable the nurse to obtain information about the disease
	2	Using mobile applications improves my clinical performance
	3	Using mobile applications can increase my productivity and efficiency
	4	With mobile applications, I can do my tasks well and at an acceptable speed
	5	Using a mobile application improves my communication with the medical staff
Perceived ease of use	6	I can easily do what I want with mobile applications
	7	I can easily use mobile applications
	8	Learning to use mobile applications is easy for me
	9	The use of mobile applications requires little mental effort
	10	Mobile applications enable me to do my job in a more comfortable way
Compatibility	11	Mobile applications are compatible with my workflow
	12	Mobile applications are proportionate to my needs
	13	Using a mobile application is *not* boring for me
	14	Mobile applications in the health domain work well with different mobile phones with different operating systems
Complicatedness	15	Mobile applications do not require specific technical skills
	16	Mobile applications do not require high mental effort
	17	Nurses can easily learn to use mobile applications
Observability	18	I can immediately see the benefits of using mobile applications
	19	The fact that the use of mobile applications in hospitals is visible in most areas encourages nurses to use these programs
	20	When nurses see other colleagues use the mobile application, the incentive to use these programs also increases
	21	It is necessary to determine the practical results and output of using mobile applications for nurses
Trialability	22	I will try mobile applications to see what they can do for me
	23	Before designing mobile applications, nurses' opinions should be considered
	24	The ability to test mobile applications for nurses is satisfactory
	25	Before deciding whether to accept a mobile application, I would like to use it as a trial
Relative advantage	26	It’s a good idea to use mobile applications to access information
	27	The advantages of using the mobile applications are more than its disadvantages
	28	I can explain why the mobile applications may be useful to me
	29	Using mobile applications has a lot of advantages for me
Usage	30	I will use mobile applications in the future
	31	I recommend others to use mobile applications

Content Validity Index (CVI) and Content Validity Ratio (CVR) were evaluated and approved for each item of the questionnaire by 10 professors of Tabriz University of Medical Sciences. To determine the reliability, the questionnaire was distributed among 30 nurses and Cronbach alpha was obtained at α = 0.9. Discriminant and convergent validity for each construct presented in Additional file 2: Tables S1, S2.

**Fig. 2:**
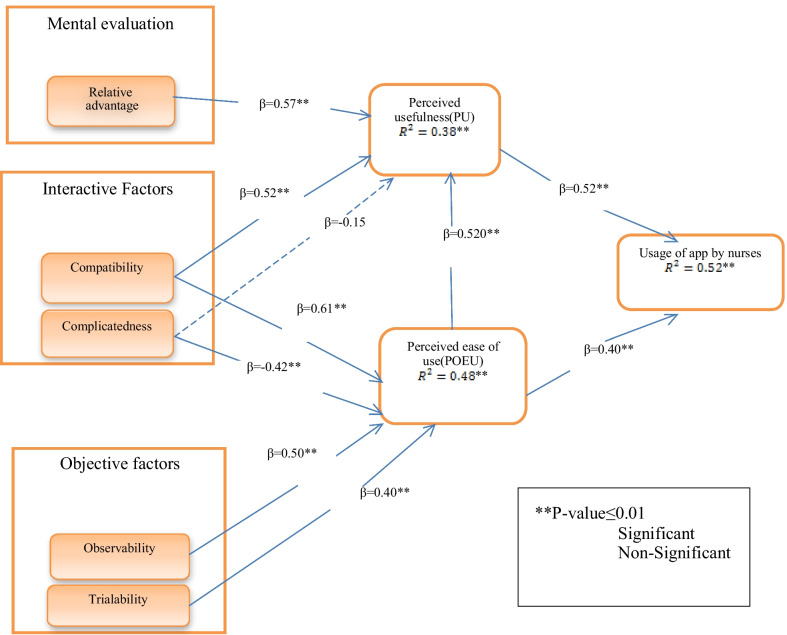
Validated proposed integrated model’s outcomes (**P value ≤ 0.01)

The questionnaires were designed and developed based on the five-point Likert scale including “totally agree”, “agree”, “neutral”, “disagree”, and “totally disagree”. Also, all methods were performed in accordance with the relevant guidelines and regulations and confirmed by experts. Data analysis was carried out using SPSS software. In order to evaluate the correlation coefficient with regard to normal distribution of data, the Pearson correlation coefficient was used. The relationship between variables was analyzed using the regression analysis test, the results of which are provided in Fig. 2.

## Results

150 nurses working at Tabriz University of Medical Sciences participated in this study, including 88.7 percent females and 10.7 percent males. 0.7 percent did not specify their gender. Regarding the level of education, the majority of 89.3% had a bachelor’s degree. The average age of participants was 32 years and the average work experience was 10 years. The participant’s demographic information is given in Table 2.

**Table 2:** Demographic information of the sample

Demographics	Category	Freq	Per	Mean
Sex	Female	133	88.7	
	Mail	16	10.7	
	Missing	1	0.7	
	Total	150	100	
Age	20$\ge$	13	8.7	32
	21–30	54	36.0	
	31–40	44	29.3	
	41–50	26	17.3	
	50$\le$	2	1.3	
	Missing	11	7.3	
	Total	150	100	
Work experience	1–5	34	22.7	10
	6–10	21	14.0	
	11–15	31	20.7	
	16–20	11	7.3	
	21–25	9	6.0	
	26$\le$	2	1.3	
	Missing	42	28.0	
	Total	150	100	
Post	Nurse	30	20.0	
	Nurse section	82	54.7	
	Head nurse	10	6.7	
	Supervisor	2	1.3	
	Matron	0	0	
	Missing	26	17.3	
	Total	150	100	

Table 3 shows the relationship between variables of the proposed model. As it is observed, there is a direct and significant relationship between TAM variables including perceived ease of use and perceived usefulness. Also, perceived ease of use and perceived usefulness have a direct and significant relationship with rate of using mobile applications by nurses. Also, there is a direct and significant relationship between relative advantage and perceived ease of use and also between compatibility and perceived ease of use and perceived usefulness. Nevertheless, complicatedness and perceived usefulness do not have a significant relationship. Other findings indicate that there is a direct and significant relationship between observability and trialability with perceived ease of use. The results of correlation analysis are given in Table 3.

**Table 3:** Results of correlation analysis between variables of the proposed integrated model

	Perceived usefulness	Perceived ease of use	Compatibility	Complicatedness	Observability	Trialability	Relative advantage	Usage
Perceived usefulness	1							
Perceived ease of use	0.520**	1						
Compatibility	0.528**	0.616**	1					
Complicatedness	0.150	0.421**	0.380**	1				
Observability	0.553**	0.500**	0.457**	0.274**	1			
Trialability	0.426**	0.405**	0.244**	0.207*	0.617**	1		
Relative advantage	0.570**	0.523**	0.504**	0.054	0.504**	0.326**	1	
Usage	0.524**	0.407**	0.406**	0.087	0.488**	0.364**	0.685**	1

**P value is significant at the 0.01 level (2-tailed)

*P value is significant at the 0.05 level (2-tailed)

The results of Fig. 2 indicate that perceived usefulness and perceived ease of use have a direct and significant effect on nurses’ use of mobile applications. Also, perceived ease of use has a direct and significant effect on perceived usefulness. Other findings of this figure suggest that relative advantage and compatibility have a direct and significant effect on perceived usefulness, while complicatedness has no significant effect. This figure also shows that compatibility, complicatedness, observability and trialability have a direct and significant relationship on perceived ease of use.

## Discussion

This study is an integration of two theories, TAM and DOI in order to identify the effective factors of using mobile health applications by nurses. When two theories of TAM and DOI are integrated, a unified framework is created for better identification and prediction of user acceptance and acceptance of innovative technology on the basis of which a better perception can be obtained regarding means of acceptance and usage of mobile applications by nurses.

The research findings indicate that there is a direct and significant relationship between perceived usefulness and rate of using mobile applications, with a standard coefficient of (*P* value ≤ 0.01) β = 0.52. Therefore, H1 is confirmed meaning that perceived usefulness has a direct and significant effect on nurses’ usage of mobile applications. These results are in line with similar findings obtained by Holdan (2010), Zhang (2008), Kowitlawakul (2008), Morton (2008) and Abdekhoda (2016) [22, 26, 37].

Also, the results of this study indicate that perceived ease of use has a direct and significant effect on mobile application use by nurses [(β = 0.40), (*P* value ≤ 0.01)]. Thus, H2 is also confirmed. These results are supported by the findings of Abdekhoda (2016), Zhang (2008) and Kowitlawalkul (2008) [22, 26, 38]. It can be inferred from the findings of the current and previous studies such as Kowitlawalkul (2008), Zhaohua (2011), Ortega Egea (2011), Pai (2010) and Zhang (2008) that perceived ease of use has a direct and significant effect on acceptance of novel technologies by users and this issue should be given more attention in the future [26, 38, 39].

With regard to H3 and the relationship between perceived ease of use and perceived usefulness, the findings suggest that perceived ease of use has a direct and significant effect on perceived usefulness [(β = 0.52), (*P* value ≤ 0.01)]. Therefore, H3 is also confirmed. Similar results are obtained in the study by Kowitlawalkul [38].

The findings of this research indicate that relative advantage has a direct and significant effect on perceived usefulness [(β = 0.57), (*P* value ≤ 0.01)], thus, confirming the fourth hypothesis H4. In addition, studies by Wu (2008), Zhang (2008), Abdekhoda (2016), Ping Yu (2009) and Conrad (2009) show that relative advantage has a significant and considerable effect on perceived usefulness [22, 26, 30, 40]. Based on these findings and other similar studies, it can be concluded that relative advantage is an important factor that directly and considerably affects the use of mobile applications.

As it is observed from Fig. 2, compatibility has a direct and considerable effect on perceived usefulness [(β = 0.52), (*P* value ≤ 0.01)]. Therefore, H5 is confirmed. In addition, the results indicate that compatibility has a direct and considerable effect on perceived ease of use [(β = 0.61), (*P* value ≤ 0.01)], which confirms the sixth hypothesis H6. This finding is in line with studies by Zhang (2008), Chew (2004), Oh (2003) Tung (2008) and Abdekhoda (2016) [22, 26, 27, 41]. Similar studies came to the conclusion that compatibility has a direct and considerable effect on perceived ease of use and perceived usefulness. Therefore, compatibility should be considered a significant factor during the use and acceptance of information systems such as compatibility with using mobile applications.

The results of this study indicate that complicatedness does not have a direct and significant effect on perceived usefulness [(β = − 0.15), (*P* value ≤ 0.01)]. As a result, H7 was not confirmed. However, other findings of this research show that complicatedness has a direct and significant effect on perceived ease of use [(β = − 0.42), (*P* value ≤ 0.01)], which confirms the eighth hypothesis. The results of this study support the findings of Oh (2004), Lee (2003), Abdekhoda (2016) and Atkinson (2007) that came to the conclusion that complicatedness has a negative and indirect effect on perceived usefulness and perceived ease of use [22, 29, 32, 41]. However, the findings of Wu (2008) indicate that complicatedness has a direct effect on the acceptance of E-CRM. When the use of a specific information system is followed by some complexities, the users tendency to work with that information systems considerably decreases [42].

As indicated in Fig. 2, the relationship between observability and perceived ease of use is significant, while observability has a direct and significant effect with [(β = 0.50), (*P* value ≤ 0.01)], thus confirming H9. This is while Sanayie (2012) and Abdekhoda (2016) indicated in their studies that observability does not have a direct and significant effect on perceived ease of use [22, 32].

Finally, with regard to the relationship between trialability and perceived ease of use, the results of this study indicate that trialability has a direct and considerable effect on perceived ease of use [(β = 0.40), (*P* value ≤ 0.01)], thus confirming H10. Chew (2004), Kendal (2001), Faiers (2007), Conrad (2009) and Sanayie (2012) have come to the conclusion that trialability has a direct and considerable effect on perceived ease of use which is of utmost significance and should be further considered [30–33].

The results of this study also show that this model describes and explains approximately 52 percent of the variance of mobile application use by nurses. In addition, the results indicate that 38% variance of perceived usefulness and 48% variance of perceived ease of use is depicted by five constructs of DOI and two key factors of TAM.

In this study, only nurses working at Tabriz University of Medical Sciences were evaluated, which may affect the generalization of the results, thus making it a limitation of the study. Secondly, this was a cross sectional study carried out in summer 2020, for which common method bias should be considered, although, Harman's single factor test show acceptable level of the total variance explained (31.936). Of course, dividing the questionnaire in two parts, independent and dependent variables; counterbalanced of the measures, and using different answering scale; was applied to minimize common method bias. In addition, data collection from the questionnaire was done by means of the nurses’ self-declarations, an issue which can be considered in future studies.

## Conclusion

As one of the key members in healthcare systems, nurses have a valuable role in teaching patients. This is because they have greater access to patients and their families and spend a great amount of time caring for them. One of the methods used today to obtain health information are mobile applications which individuals use due to easy access anytime and anyplace. Theoretically, the current study clearly identified effective factors in nurses’ use of mobile health applications based on the integrated model of TAM and DOI. Perceived usefulness, perceived ease of use, relative advantage, compatibility, trialability, and observability are factors effecting nurses’ use of mobile health applications. Also, the findings show that the integrated model of TAM and DOI is an appropriate conceptual model to elucidate nurses' intentional behavior in adoption of mobile health applications.

Practically, this study recommended that designers of mobile applications consider the above determinant criteria in the design and framework of their programs in order to obtain greater user satisfaction. Based on the findings of this study, in using mobile applications by nurses, complicatedness in designing mobile applications negatively affected perceived usefulness and perceived ease of use. This is while trialibility and the compatibility with nurses and users will increase the use of mobile applications.

## Supplementary Information

**Additional file 1**. Convergence and Discriminant Validity Results.

**Additional file 2**. Additional information of pervios studies.

## Data Availability

The datasets used and/or analyzed during the current study are available from the corresponding author on reasonable request.
