# Supplementary material for: Determinant factors in adopting mobile health application in healthcare by nurses
Source: BMC Med Inform Decis Mak. 2022 Feb 22;22:47. doi: 10.1186/s12911-022-01784-y (PMC8862523; doi:10.1186/s12911-022-01784-y)
Supplement: Supplementary file 1 — Additional file 1. Convergence and Discriminant Validity Results. [file 12911_2022_1784_MOESM1_ESM.docx]

Supplementary file

For example, in studies carried out by O’Connor et al. (2018) the numerous advantages of smartphone technology such as better access to educational content, enhanced knowledge and self-confidence and reducing stress level while learning have been reported by nursing students. Obstacles such as negative views of the nursing staff, lack of Wi-Fi connection, and quality of educational content existing in practical smartphone applications have been reported as some of the issues that prevent the acceptance of learning to work with smartphones in clinical nursing education (5).

In their study, Hsu et al. (2018) stated that smartphone applications are an effective learning tool and assist in transferring knowledge to nursing students and these programs can enhance nurses’ experience (6).

In a study by Mazaheri et al. (2017), results indicated the educational needs of interns in the realm of identifying and using medications in the clinical setting. The application of medication software by means of smartphones as integrated with regular education in the clinical setting was effective in cognitive dimensions and medication error behaviors of nursing students (7).
