# Supplementary material for: Determinant factors in adopting mobile health application in healthcare by nurses
Source: BMC Med Inform Decis Mak. 2022 Feb 22;22:47. doi: 10.1186/s12911-022-01784-y (PMC8862523; doi:10.1186/s12911-022-01784-y)
Supplement: Supplementary file 2 — Additional file 2. Additional information of pervios studies. [file 12911_2022_1784_MOESM2_ESM.docx]

| Construct | Item number | Factor loading | Cronbach’s  Alpha | Average  Variance  Extracted  (AVE) |
| --- | --- | --- | --- | --- |
| Perceived  Usefulness |  |  | 0.901 | 0.522 |
|  | 1 | 0.777 |  |  |
|  | 2 | 0.562 |  |  |
|  | 3 | 0.653 |  |  |
|  | 4 | 0.523 |  |  |
|  | 5 | 0.502 |  |  |
| Perceived  ease of use |  |  | 0.716 | 0.501 |
|  | 6 | 0.725 |  |  |
|  | 7 | 0.622 |  |  |
|  | 8 | 0.596 |  |  |
|  | 9 | 0.785 |  |  |
|  | 10 | 0.759 |  |  |
| Compatibility |  |  | 0.826 | 0.512 |
|  | 11 | 0.516 |  |  |
|  | 12 | 0.501 |  |  |
|  | 13 | 0.629 |  |  |
|  | 14 | 0.639 |  |  |
| Complicatedness |  |  |  |  |
|  | 15 | 0.985 | 0.856 | 0.516 |
|  | 16 | 0.625 |  |  |
|  | 17 | 0.548 |  |  |
| Observability |  |  | 0.986 | 0.520 |
|  | 18 | 0.625 |  |  |
|  | 19 | 0.698 |  |  |
|  | 20 | 0.619 |  |  |
|  | 21 | 0.599 |  |  |
| Trialability |  |  | 0.746 | 0.513 |
|  | 22 | 0.532 |  |  |
|  | 23 | 0.562 |  |  |
|  | 24 | 0.569 |  |  |
|  | 25 | 0.542 |  |  |
| Relative  Advantage |  |  | 0.877 | 0.511 |
|  | 26 | 0.785 |  |  |
|  | 27 | 0.763 |  |  |
|  | 28 | 0.721 |  |  |
|  | 29 | 0.456 |  |  |
| Usage |  |  | 0.956 | 0.521 |
|  | 30 | 0.832 |  |  |
|  | 31 | 0.816 |  |  |

Supplementary Table1. Convergence Validity Results

Supplementary Table2. Discriminant Validity

| Usage | Relative advantage | Trialability | Observability | Complicatedness | Compatibility | Perceived ease of use | Perceived usefulness |  |
| --- | --- | --- | --- | --- | --- | --- | --- | --- |
|  |  |  |  |  |  |  | **0.756** | Perceived usefulness |
|  |  |  |  |  |  | **0.712** | 0.399 | Perceived ease of use |
|  |  |  |  |  | **0.796** | 0.258 | 0.321 | Compatibility |
|  |  |  |  | **0.702** | 0.396 | 0.358 | 0.235 | Complicatedness |
|  |  |  | **0.723** | 0.378 | 0.289 | 0.367 | 0.368 | Observability |
|  |  | **0.738** | 0.306 | 0.351 | 0.294 | 0.289 | 0.389 | Trialability |
|  | **0.716** | 0.394 | 0.361 | 0.364 | 0.378 | 0.397 | 0.347 | Relative advantage |
| **0.716** | 0.309 | 0.328 | 0.259 | 0.360 | 0.396 | 0.352 | 0.361 | Usage |
